# Molecular Mechanism of Radioresponsiveness in Colorectal Cancer: A Systematic Review

**DOI:** 10.3390/genes15101257

**Published:** 2024-09-26

**Authors:** Matthew Y. H. Lau, Md Zahirul Islam Khan, Helen K. W. Law

**Affiliations:** 1 Department of Health Technology and Informatics, Faculty of Health and Social Science, The Hong Kong Polytechnic University, Hunghom, Hong Kong, China; 21018218g@connect.polyu.hk; 2 Department of Biological Sciences, University of Texas at El Paso, El Paso, TX 79968, USA; mislamkhan@utep.edu

**Keywords:** colorectal cancer, radioresponse, biomarkers, molecular mechanisms, gene mutation

## Abstract

**Background/Objectives**: Colorectal cancer (CRC) is the third most diagnosed cancer globally. Radiotherapy is a common treatment strategy for patients but factors such as gene expressions and molecular mechanism effects may affect tumor radioresponse. The aim of this review is to systematically identify genes suggested to have molecular mechanism effects on the radioresponsiveness of CRC patients. **Methods**: By following the PRISMA guidelines, a comprehensive literature search was conducted on Pubmed, EMBASE and Cochrane Library. After exclusion and inclusion criteria sorting and critical appraisal for study quality, data were extracted from seven studies. A gene set analysis was conducted on reported genes. **Results**: From the seven studies, 56 genes were found to have an effect on CRC radioresponsiveness. Gene set analysis show that out of these 56 genes, 24 genes have roles in pathways which could affect cancer radioresponse. These are *AKT1*, *APC*, *ATM*, *BRAF*, *CDKN2A*, *CTNNB1*, *EGFR*, *ERBB2*, *FLT3*, *KRAS*, *MET*, *mTOR*, *MYC*, *NFKB1*, *KRAS*, *PDGFRA*, *PIK3CA*, *PTEN*, *PTGS1*, *PTGS2*, *RAF1*, *RET*, *SMAD4* and *TP53*. The current project was conducted between the period May 2024 to August 2024. **Conclusions**: The current review systematically presented 56 genes which have been reported to be related to RT or CRT treatment effectiveness in rectal cancer patients. Gene set analysis shows that nearly half of the genes were involved in apoptosis, DNA damage response and repair, inflammation and cancer metabolism molecular pathways that could affect cancer radioresponse. The gene cohort identified in this study may be used as a foundation for future works focusing on the molecular mechanism of specific pathways contributing to the radioresponse of CRC.

## 1. Introduction

Colorectal cancer (CRC) is a cancer developed in the colon and/or rectum. According to the statistics, an estimated number of 1,880,725 patients consisting of 1,148,515 colon cancer and 732,210 rectal cancer cases were diagnosed in 2020, making CRC the third most diagnosed cancer worldwide [1]. Based upon aging projectiles, population growth and human development, researchers have predicted that by 2040, new CRC cases could reach to 3.2 million, thus prompting the Government to take serious implementations in early screening and detection programs [2,3,4].

In order to achieve a high cure rate, CRC should be screened and detected as early as possible, which could be achieved by a number of different methods. Usually, medical imaging techniques such as sigmoidoscopy or colonoscopy can be performed to search the sigmoid, colon and rectum for abnormal growths or polyps, which could be removed and biopsied for more detailed further analysis.

If detected early, CRC can have a high curative rate. When a person is exhibiting symptoms and abnormal screening results, diagnostic medical assessments, which usually consist of blood and imaging tests, should be performed to determine whether the CRC diagnosis is positive or not [5]. During diagnosis, cross-sectional imaging tests would be conducted to allow the clinician to assess the severity, location and size of the cancer so that surgical options available could be planned. Biopsies would be sent to the lab to determine malignancy by biomedical tests such as histopathological examination of cell characteristics or molecular analysis of gene mutations and protein markers [6].

After diagnosing CRC, the next step is to determine the extent of locoregional and distant spread. Similar to other types of solid tumor cancers such as bladder cancer and breast cancer, the assessment usually comes in the form of a tumor, node, metastasis (TNM) classification [7,8]. Once a patient’s TNM values have been accessed, the overall stage could be determined by combining the categories and other factors together. This is commonly known as group staging [9]. Other classification systems, such as Astler–Coller classification and Full Dukes classification, both originating from the Dukes classification have also been reported [10,11].

CRC treatment options and recommendations depend on a number of factors including cancer severity, treatment side effects, the patient’s preferences and the overall health of the patient. Generally, surgery, radiotherapy and chemotherapy are the main treatments. Frequently, radiotherapy and chemotherapy are combined together as chemo-radiotherapy (CRT) and administered to the patient either before surgery to give neoadjuvant CRT (nCRT) or after surgery, as adjuvant CRT [12].

Measuring the shrinkage of the tumor size by imaging methods such as CT-scans is one of the most direct reflections for preoperative treatment results in patients before surgery. Once resection is performed, the specimen could be sent to the lab for further analysis to provide assessment on treatment efficiency and effects. When evaluating the response of neoadjuvant therapies and predicting prognosis, a grading system called Tumor Regression Grade (TRG) could be used. TRG provides a grade on the histology of the resected tumor to deduce post-therapeutic pathological information such as whether TNM downstaging could be observed, signifying an improved cancer stage.

For CRC patients, since surgery is the common and first-line treatment for colon cancers, TRG assessment usually just applies to rectal cancers and especially to locally advanced rectal cancer (LARC) patients often undergoing nCRT. There are a few TRG systems currently in used with the most widely accepted being Mandard, Dworak and AJCC [13].

Although all strategies discussed here aim to completely kill off cancer cells and minimize the harm caused by the tumor, many dependents could affect treatment outcome. One example is the treatment response of CRC patients to ionizing radiation (radioresponsiveness) and the underlying molecular mechanism affecting it, such as gene variants and differential expressions.

Thus, by studying the published literature over the past decade, the current review aims to systematically identify molecular mutations suggested to have effects on the radioresponsiveness of CRC patients.

The clinical implication of the current study is to provide an understanding in molecular mechanisms which can affect radioresponsiveness of CRC patients under RT to help clinicians personalize and select the most effective treatment options through methods such as companion therapies. Furthermore, the current review highlights the importance of considering genetic variants and expression differences in CRC patients receiving radiation therapy and provides a foundation for future research in this area.

The question of interest in PICO(T) format is as follows: What molecular mechanisms (C) were suggestive to have an effect on the radioresponsiveness (O) of CRC patients receiving RT or CRT (P), when compared with the unaltered molecular mechanism (I) within the past decade from 2012 to 2024 (T).

## 2. Materials and Methods

This systematic review was registered in Open Source Framework registries (10.17605/OSF.IO/PSJ79) and follows the PRISMA 2020 (Preferred Reporting Items for Systematic Reviews and Meta-Analyses) guidelines [14].

### 2.1. Search Strategy

A comprehensive literature search was performed with Pubmed, EMBASE and Cochrane Library. In order to obtain the highest relevant results possible, the following keywords were used: ‘Colorectal cancer’ OR ‘Colon cancer’ OR ‘Rectal cancer’ AND ‘Radiotherapy’ AND ‘Radiosensitivity’ AND ‘Genes’. No language filters were applied; however, publication times were restricted to only the past decade from 1 January 2012 to 30 May 2024.

### 2.2. Inclusion and Exclusion Criteria

The inclusion and exclusion criteria for studies to be included in this review were set based on the PICO(T) question of interest. Inclusion criteria were observational cohorts of all designs; studies with patients diagnosed with primary CRC by pathological confirmation; studies with CRC patients undergoing either RT or CRT as a neoadjuvant or main treatment; and studies evaluating genes related to CRC radiosensitivity based on RT or CRT treatment response. Exclusion criteria included publications before 1 January 2012 and after 30 May 2024; studies focused solely on either chemotherapy, immunotherapy and/or surgical outcomes; duplicated studies and/or incomplete data sets; conference abstracts, reviews or theses; no comparison with normal tissue genes or pretreatment data; non-clinical studies or no outcome data on tumor responses to either RT or CRT and studies associated with diseases other than CRC

The PRISMA flow chart provided as Figure S1 of Supplementary Materials illustrates the search and selection strategy for this review [14]. The initial search produced 367 results from Pubmed (n = 108), EMBASE (n = 247) and Cochrane Library (n = 12) together. A total of 300 results remained for abstract and title screening after the removal of duplicates (n = 65) and retractions (n = 2). Study screening was performed independently by all three authors according to the study inclusion and exclusion criteria.

### 2.3. Study Screening

Initial abstract and title screening excluded 242 studies comprising the following exclusion reasons: studies focused solely on either chemotherapy, immunotherapy and/or surgical outcomes (n = 13), conference abstracts, reviews or theses (n = 105), no comparison with normal tissue genes or pretreatment data (n = 10), non-clinical studies or no outcome data on tumor responses to either RT or CRT (n = 94) and studies associated with diseases other than CRC (n = 20). A total of 58 citations were exported back onto Endnote for full text retrieval, of which 3 studies were inaccessible, 19 were either conference abstracts, reviews or theses, 2 focused solely on either chemotherapy, immunotherapy and/or surgical outcomes, 22 were non-clinical studies or no outcome data on tumor responses to either RT or CRT, 3 publications were associated with diseases other than *CRC* and 2 had no comparison with normal tissue genes or pretreatment data. A total of 5 additional reports were identified by citation screening; however, none of them were included as 3 were unavailable and 2 were excluded for non-clinical studies or no outcome data on tumor responses to either RT or CRT. In the end, 7 studies were found to have satisfy the criteria to be included (Figure S1).

### 2.4. Quality Assessment Protocol

In total, 7 studies were identified to undergo critical appraisal assessment. These were Jiang et al. [15], Zhu et al. [16], Molinari et al. [17], Senetta et al. [18], Ha et al. [19], Dzhugashvili et al. [20] and Gantt et al. [21]. The publication qualities of the eligible studies were assessed by the National Institute of Health (NIH) Study Quality Assessment Tool for Observational Cohort and Cross-Sectional studies [22]. According to the assessment criteria, each publication will be given a point depending on the evidence to answer the criteria’s question. A “Yes” with 1 point will be awarded if sufficient evidence was shown in the study, whilst a “No” will be allocated where there is not enough information and be provided with 0 points. If the question is not applicable or irrelevant to appraise for in the current review, a “Not Applied” will be granted with no points to score. For each study, the scores will count together towards a final grade which will represent either good quality (10–14 points), satisfactory quality (6–9 points) or bad quality (0–5 points). Critical appraisal was performed by all three authors individually to avoid bias.

### 2.5. Data Extraction and GSEA

Key components deemed significant were extracted from the included studies including study characteristics, clinical characteristics and outcome measurements. If possible, the original authors were contacted to request or verify any data required. For study characteristics, the general and background information of the study were extracted including publication year, study country, recruitment criteria, sample size, study age and gender size. Clinical characteristics including pathological information containing pre-therapeutic staging, tumor histological grading and other information (such as CEA levels) were extracted together with the treatment protocol of the study. Outcome measurements consisting of Tumor responses and mutation information were abstracted. Tumor response parameters include the TRG grade and criteria applied as well as post-therapeutic staging. For mutation information, the gene of interest, mutation type and frequency of mutation were reported if available in each study. The method of mutation detection was also recorded. Any other additional information was noted under the remark section.

In order to delve more on the relationship between genes and CRC radioresponsiveness (Table S1), the genes of interests reported by each study were submitted onto the National Center of Biotechnology Information (NCBI) gene search to obtain information consisting of ENSG ID, full nomenclature, chromosome location and brief function (Table S2). Furthermore, the data obtained from NCBI were uploaded onto two online bioinformatics platforms for gene set enrichment analysis (GSEA) at Enrichr and WebGestralt to predict how molecular mechanisms were involved in regard to radiosensitivity [23,24,25].

## 3. Results

### 3.1. Quality Assessment Results

Out of the seven studies, only Senetta et al. [18] and Ha et al. [19] provided justifications for their sample size. However, as suggested by the assessment tool, this criterion does not inflict a “fatal flaw” since the participant size is not essential in answering the research question of the study. Study blinding was also not necessary since there was no need for masking parameters for investigators and prior information, and measures must be known in order to deduce the effectiveness of RT/CRT treatment leading to the discovery of genes of interest. Nevertheless, all seven studies [15,16,17,18,19,20,21] displayed relevant publication quality in satisfying the NIH assessment tool by scoring a total score above 10 (Table 1) and were considered eligible to be further analyzed.

### 3.2. Study Characteristics

Table 2 shows the study characteristics extracted from the seven eligible studies. In summary, a total of 691 CRC patients consisting of 459 males and 232 females were included in the current systematic review. The publication year ranged from 2013–2019 with two studies conducted in China and Italy and one study each from Republic of Korea, Spain and the USA. The majority of the study subjects are middle aged with the mean age being >50 years.

Patient recruitment criteria show that all studies investigated only on patients diagnosed with rectal cancers. Three studies [17,18,20] focused specifically into locally advanced rectal cancers (LARCs). Exclusion criteria for genetic analysis were reported in five studies [15,16,17,18,20]. All the patients recruited were eligible for nCRT.

In order to compare the differences between therapy outcomes, Ha et al. [19] studied two cohorts.

### 3.3. Clinical Characteristics

In terms of pathological information, only Senetta et al. [18] did not report pre-therapeutic staging in their study. The rest of the studies either reported pre-therapeutic TNM stages [16,17,20], clinical stages [19,21] or both [15], according to the AJCC or NCCN criteria. In summary, 21 patients were diagnosed at an early stage with either T0–T2 pre-therapeutic TNM staging or clinical stage I, and a total of 585 patients with intermediate or late stage cancer corresponding to T3–T4b or clinical stage II–IV. According to available data for N-stage grouping, 107 patients had no regional lymph node metastasis (N0), while 243 patients had some degree of lymph node spreading (N+–N2). Regarding metastasis M stage, only Zhu et al. [16] reported 122 patients with no distant metastasis (M0) and 26 with metastasis to distant organs beyond regional lymph nodes (M1). The rest of the studies did not provide any data on the M stage of their patients (Table 3).

All studies, except Molinari et al. [17], provided histological information on the tumor section. A three-tier grading scheme was applied by Jiang et al. [15], Senetta et al. [18] and Dzhugashvili et al. [20], while Zhu et al. [16], Ha et al. [19] and Gantt et al. [21] used differentiation levels to grade their tumors. In total, 385 patients exhibit either low to intermediate (G1–G2) or well to moderately differentiated tumor grades, whilst high-grade (G3) and poorly or undifferentiated tumors consisted of 103 patients (Table 3).

Tumor location was briefly described in two studies [16,20]. Zhu et al. [16] also provided serum CEA levels along with Ha et al. [19]. Jiang et al. [15] reported the carcinoma type their patients possessed, whilst Gantt et al. [21] reported the presence of differential vascular and lymphatic invasions.

All patients in the studies received nCRT and the detailed treatment protocol consisting of chemotherapy and radiotherapy dosages and surgery performed, which were extracted from each publication. Chemotherapy regimen consisting of either capecitabine (Xeloda) and/or 5-fluorouracil (5-Fu) were given to all patients. Oxaliplatin was administered as a XELOX cocktail in four studies, out of which three patients were given it as a neoadjuvant [15,16,18]. In Jiang et al.’s study, patients received XELOX and mFOLFOX-6 cocktail cycles before beginning an RT regimen. For Dzhugashvili et al.’s study, patients exhibiting adverse risk factors were infused with either a XELOX or FOX cocktail as an adjuvant chemotherapy after surgery [20]. Standard radiation therapy ranging from 45–50 Gy was given in fractions of 25–30 for the majority of the studies. Surgery by either TME, radical or resection approach was performed on the patients, ranging from 4 to 10 weeks after the completion of nCRT as reported by each respective study (Table 3).

### 3.4. Outcome Characteristics

Table 4 outlines the extracted outcome measurements from corresponding studies. Zhu et al. [16] and Molinari et al. [17] both used Dwoark criteria for TRG assessment, whilst Senetta et al. [18] and Ha et al. [19] used the Mandard criteria. Jiang et al. [15] applied the NCCN method and Gantt et al. [21] scored with the AJCC system. Instead of TRG grading, Dzhugashvili et al. [20] reported the response by measuring differences in tumor sizes before and after the treatment. Brief definition of TRG grades were extracted and provided in the remark section of Table 4.

In terms of mutation information, both genetic variants and gene expression differences were found to have an effect on radioresponse in CRC patients. Most studies engaged in sequential mutation information such as point mutations and polymorphisms. Zhu et al., Molinari et al. and Dzhugashvili et al. reported alterations at the gene expression level [16,17,20]. Impact of post-transcriptional regulation by microRNA (miRNA) cluster expressions was reported by Molinari et al. [17]. Only Senetta et al. explored the post-transcriptional response at the protein expression level [18]. At the epigenetic regulation level, Ha et al. is the only study to describe the effects of gene silencing by hypermethylation of CpG islands resulting in downgrade observed in patients [19].

Gantt et al. [21] and Ha et al. [19] did not report post-therapeutic TNM stages, whilst the remaining five studies reported the post-therapeutic pathological TNM stage after surgical resection of the tumor. In total, 175 patients were reported to have achieved downstaging by Jiang et al., Zhu et al. and Senetta et al. [15,16,18]. As downstaging was not observed or reported by Molinari et al. and Dzhugashvili et al., direct comparison with pre-therapeutic staging information (Table 3) was performed [17,20]. More patients were observed in the T0, T1 and N0 stage and less patients in the T3 stage for Molinari et al.’s study, whilst Dzhugashvili et al.’s study shows a great decline of patients having T3–4 stage, from 150 to 31 [17,20] (Table 4).

### 3.5. GSEA

The 56 genes with an effect on radioresponse were uploaded onto Enrichr and WebGestalt for gene set enrichment analysis. The result shows that 24 out of the 56 genes have roles in pathways which could affect cancer radioresponse (Table 5). These genes are *AKT1*, *APC*, *ATM*, *BRAF*, *CDKN2A*, *CTNNB1*, *EGFR*, *ERBB2*, *FLT3*, *KRAS*, *MET*, *mTOR*, *MYC*, *NFKB1*, *KRAS*, *PDGFRA*, *PIK3CA*, *PTEN*, *PTGS1*, *PTGS2*, *RAF1*, *RET*, *SMAD4* and *TP53*. This suggests that mutation to these genes will substantially alter the function and effectiveness of the corresponding pathway, which affects how a cancer responds to cancer treatments such as RT.

The main pathways outlined were apoptosis, DNA damage response and repair, inflammation and cancer metabolism pathways (Table 5). The remaining genes either have roles in non-significant pathways or produced inconclusive results. It should be noted that *AKT1*, *KRAS*, *NRAS* and *PIK3CA* all have roles in all four pathways followed by *RAF1* and *PTEN* with three functions each. Genes covering two pathways include *CDKN2A*, *EGFR*, *ERBB2*, *MYC*, *NFKB* and *TP53.* There are 15 genes and 12 genes involved in cancer metabolism pathways and DNA damage response, respectively. A total of 10 genes belong to the Inflammation pathway, and 9 genes were involved in the apoptosis pathway.

Gene set enrichment analysis from the Enrichr and WebGestalt online platforms show that 24 genes identified from reviewed studies have functions in at least one of the main cancer pathways. Notably, more than half (15 genes) are related to cancer metabolism.

## 4. Discussion

The current systematic review aims to explore the question on what molecular mechanisms were suggestive to have an effect on the radioresponsiveness of CRC patients receiving RT or CRT, based on publications in the past decade (from 2012 to 2024). Seven publications were considered admissible in providing an insight to the study aim.

Molecular diagnostics have been the recent trend for disease diagnosis and precision treatments. By screening and identifying specific molecular mechanisms, the genetic profile of an individual could be reported leading to a more personalized care. This aspect is particularly important in CRC as there is currently no one single best treatment due to the divergence in tumor genetics. A total of 56 genes were identified to influence radioresponse at the molecular level by genetic variants (n = 27) and gene expression differences (n = 29) (Table S1).

Both gene variants and gene expression differences can be regarded as molecular mechanisms as they can directly affect cellular processes and functions at molecular levels.

Gene variations such as polymorphisms and point mutations are caused by differences in gene sequences which can occur in both coding and non-coding regions, influencing the regulation of gene expressions. For example, a variant that results in a change in an amino acid in a protein might alter the protein’s function, potentially leading to a disease state or altering the response to a treatment like radiotherapy.

On the other hand, differences in gene expression refer to the variability in the level at which different genes are transcribed and translated into proteins in cells under different conditions. These could be controlled by various factors such as epigenetic modifications or post-translational regulations. Changes in gene expression can alter cellular functions and responses to stimuli, including response to treatments such as radiotherapy.

**Figure 1:**
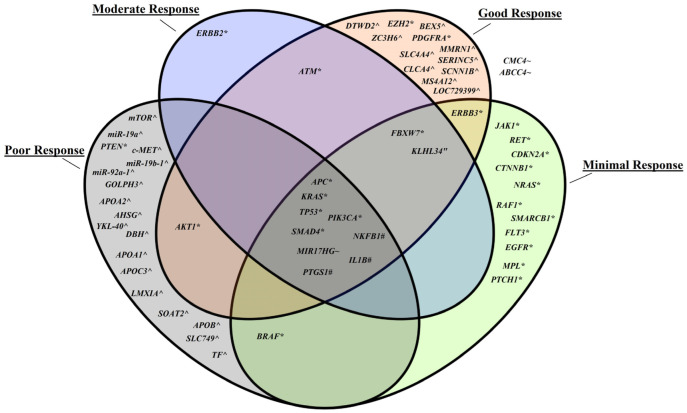
Association of genes to CRC radioresponse. (*) illustrates gene variants by point mutations or deletions; (^) describes upregulation or high expression; (~) denotes copy number alterations; (“) means CpG site hypermethylation and (#) shows polymorphisms.

In the context of radioresponse in CRC patients, certain gene variants might make the tumor cells more resistant to radiation by enhancing cellular pathways such as DNA damage response and repair and apoptosis. Similarly, differences in the expression of certain genes might influence the effectiveness of radiotherapy by altering the cellular response to radiation-induced damage such as the cancer metabolism and inflammation pathways. Therefore, both gene variants and gene expression differences can be considered molecular mechanisms as they can directly impact actions and traits of cells at the molecular level, including their response to treatments [26]. A summary of our findings of identified genes in relation to radioresponse is illustrated below (Figure 1).

### 4.1. Potential Molecular Biomarkers for Radioresponse

Within our gene cohort, several genes have already been extensively studied and are currently well recognized as molecular biomarkers, therapeutic targets and prognosis predictors. Specifically for CRC, the somatic mutations of gene *BRAF*, *KRAS*, *NRAS* and PIK3CA are currently being used as routine diagnostic molecular biomarkers to anticipate responses to anti-cancer treatments such as EGFR inhibitor drugs (good response with wildtype *BRAF*, *KRAS* and *NRAS*) and postoperative aspirin therapy (good response in mutation of PIK3CA) [27,28]. An example is the routine testing of *KRAS* to identify metastatic CRC patients for anti-EGFR antibody therapy [29].

Other than using gene mutations as biomarkers, some diagnostic reports would also include miRNA levels to examine treatment efficacy and disease stage, such as resistance to the FOLFOX regimen in CRC patients with up regulated *miR-19a* and elevation of *miR-92a* in advanced CRC [30,31]. In our review, although there was no significant differences, non-responders exhibited higher gene expression variability of *miR-19a*, *miR-19b-1* and *miR-92a-1* levels. Interestingly, Sun et al. recently demonstrated that *miR-19b* had an inverse relationship with the tumor suppressor protein FBXW7and regulates the Wnt/β-catenin pathway to promote cancer stemness properties. With CRC cell lines and mice models, they also show that *miR-19b* inhibitors conveyed enhanced radiation sensitivity [32].

Epigenetic instability, characterized by the CpG island methylator phenotype (CIMP), has been recognized as a distinct cancer subgroup for over a decade. DNA hypermethylation in CpG-rich promoters represents a subgroup of CRC and provides a promising biomarker for the chemotherapy treatment forecast for 5-FU in stage III colon cancers [33,34]. The reporting of CIMP positive or negative status in CRC patients, especially with BRAF mutations, has been recommended in the reporting guideline for molecular biomarkers established by an experts panel collaborated on by the American Society for Clinical Pathology (ASCP), College of American Pathologists (CAP), Association for Molecular Pathology (AMP), and American Society of Clinical Oncology (ASCO) [35].

Currently, many biomarkers are focused on the treatment responses to anti-cancer drugs such as chemotherapy. Biomarkers reflecting cancer RT responses, such as those of CRC, are limited to only a handful, and our gene cohort could provide a mining site to dig further for exploration of biomarkers for RT response.

### 4.2. Gene Set Analysis Suggests the Potential Molecular Mechanisms of Pathways Affecting Radioresponsiveness

Multiple biological processes are associated with CRT response, giving rise to a diverse and vast connection of molecular pathways. CRT terminates cancer mainly by either inhibiting cancerous cells’ growth and preventing cell proliferation or causing tumor DNA damage and promoting cell death. With this in mind, it is reasonable to assume that apoptosis, DNA damage response and repair, inflammation and cancer metabolism pathways are significantly associated with the therapy response.

Our gene set analysis demonstrated that nearly half of the genes in our cohort have functions in one of the four mentioned pathways. Notably, the pathway with the most genes covered is cancer metabolism, followed by DNA damage response and repair. Recent studies have shown that ionizing radiation could induce tumor cellular stress, causing alterations in cancer metabolism, which could affect the susceptibility of the tumor to radiation damage [36,37]. Upon exposure to external stimuli, metabolism pathways could be altered, causing activation or suppression of different cellular functions. Autophagy is an important pathway which could affect the survival of a cancer cell. It has been reported that radiosensitivity increased in CRC cells after autophagy-related genes are knocked down, such as Atg12 inhibition by miR-214 [38,39]. A recent review has also suggested that the PI3K/AKT/mTOR pathway, hypoxia and glucose metabolism could be other potential radioresistance targets [40].

The relationship between radioresistance and the DNA damage response and repair pathway has also been broadly studied in the past decades [41]. DNA damage response and repair is one of the causes for genomic instability, which is a cancer hallmark [40]. By evading DNA damage response and repair, mutations could arise, leading to uncontrollable growth of malignant cells and ultimately to cancer. RT aims to correct this by delivering radiation-based DNA damage to tumor cells and reducing their survival. In order to proliferate, tumor cells undergo genetic modifications to increase their radioresistance.

Similar to DNA damage response and repair, apoptosis is a very popular topic for cancer researchers, and its induction is a principle aim for most anti-cancer therapies, including RT [42]. This molecular mechanism has a very prominent role in safeguarding both our micro- and macro-body environment. Regulations are critical and must be kept tightly so that any unwanted cells can be destroyed before causing any harm to the body. Recent studies have shown that radiosensitivity can be increased by modulating and arresting cell cycle checkpoints, promoting cell apoptosis to be triggered [43,44]. An example is shown in in vitro experiments with the CRC cell line HCT116, which have demonstrated that treatment with purvalanol (a cyclin-dependent kinase which could cause G2/M cell cycle arrest) can induce an early autophagic response followed by apoptosis [45,46]. The interplay of autophagy and apoptosis can also be noted in CRC cells with defective autophagy, whereas exposure with radiation causes escalation of the DNA damage response and subsequent apoptosis enhancement [46,47].

Lastly, inflammation-induced radioresistance has been previously reported in vitro with lung and breast cancer cell lines [48,49]. To our current understanding, inflammation has been recognized as an important tumorigenesis driver and the seventh cancer hallmark by linking with genetic instability [50]. Prolonged inflammation of the bowel could cause complicated conditions known as inflammatory bowel disease (IBD) and patients with IBDs (such as Crohn’s disease) have a significantly increased risk for developing CRC. Inflammation-induced epigenetic changes were suggested to initiate tumorigenesis by interaction with the tumor microenvironment and supplying growth factors and pro-inflammatory cytokines [51].

Although not outlined as a key regulatory gene in our gene analysis, NFKB has been seen as an important mechanistic link between inflammation and cancer [52,53,54]. DNA damage and mutation could arise from reactive oxygen species (ROS) and reactive nitrogen species (RNS) produced by NFKB-induced inflammation [55]. By inhibiting or altering NFKB signaling, pro-inflammatory genes were found to have decreased. Cell line experiments demonstrated that this phenomenon of reduced inflammatory activity increases autophagosome actions leading to autophagy induced apoptosis, whilst clinical studies have reported the increase of inflammatory responses in Crohn’s disease patients due to miRNA autophagy inhibition [56,57]. This suggests that inflammatory responses by the inflammation pathway could be a negative regulator for programmed cell death mechanisms such as autophagy-induced apoptosis.

### 4.3. Limitations and Improvements

There were several limitations and improvements worth considering for future works. Despite the fact that we have shortlisted the genes that may be related to radioresponsiveness, there is a lack of retrospective studies to verify the findings. In addition, all studies included here investigated solely on rectal cancer; therefore, further experimental studies are required to unravel the knowledge about the effect of gene mutation and therapy effect on colon cancer patients. The lack of colon cancer information could be due to the reason of surgery being the main treatment. Further research is required to exclusively deduce the treatment response of RT without chemotherapy intervention as CRT is the only neoadjuvant therapy reported by each study.

In our current review, a meta-analysis was not conducted for several reasons. Meta-analyses are typically used to synthesize outcome data from multiple studies to provide an estimation of treatment effects, such as survival rates or disease progression. The objective of this review was to provide a broad overview of the molecular mechanisms related to radioresponse by identifying gene mutations and differential expressions that influence the response of CRC patients to RT/CRT. Given that our main intervention was on the molecular mechanisms rather than the treatment effects, the primary concern was whether there was any pathological response to the therapy.

Furthermore, meta-analysis requires low heterogeneity between studies to provide a confident effect estimation. The focus of our review can be addressed through a variety of study designs which poses a challenge in finding a sufficient number of studies exhibiting the homogeneity necessary for inclusion in a meta-analysis. Consequently, we believe that a meta-analysis may not be the most appropriate approach for synthesizing our data.

## 5. Conclusions

The current review systematically presented 56 genes which have been reported to be related to RT or CRT treatment effectiveness in rectal cancer patients. Gene set analysis shows that nearly half of the genes are involved in molecular pathways which may affect cancer radioresponse. The gene cohort identified may be used as a foundation for future works focusing on the molecular mechanism of specific pathways contributing to the radioresponse of CRC, such as autophagy.

## Figures and Tables

**Table 1:** Critical appraisal of publication quality of eligible studies based on 13 criteria by 14 questions from the NIH assessment tool for observational cohort and cross-sectional studies.

Quality Assessment Tool for Observational Cohort and Cross-Sectional Studies								
Quality Assessed Based on:		Study						
Criteria assessed	Question to satisfy	[15]	[16]	[17]	[18]	[19]	[20]	[21]
Research Questions	1. Was the research question or objective in this paper clearly stated?	✓	✓	✓	✓	✓	✓	✓
Study population	2. Was the study population clearly specified and defined?	✓	✓	✓	✓	✓	✓	✓
	3. Was the participation rate of eligible persons at least 50%?	✓	✓	✓	✓	✓	✓	✓
Groups recruited from the same population and uniform eligibility criteria	4. Were all the subjects selected or recruited from the same or similar populations (including the same time period)? Were inclusion and exclusion criteria for being in the study prespecified and applied uniformly to all participants?	✓	✓	✓	✓	✓	✓	✓
Sample size justifications	5. Was a sample size justification, power description, or variance and effect estimates provided?	✗	✗	✗	✓	✓	✗	✗
Exposure assessed prior to outcome measurement	6. For the analyses in the given paper, were the exposure(s) of interest measured prior to the outcome(s) being measured?	✓	✓	✓	✓	✓	✓	✓
Sufficient timeframe to see an effect	7. Was the timeframe sufficient so that one could reasonably expect to see an association between exposure and outcome if it existed?	✓	✓	✓	✓	✓	✓	✓
Different levels of the exposure interest	8. For exposures that can vary in amount or level, did the study examine different levels of the exposure as related to the outcome (e.g., categories of exposure, or exposure measured as continuous variable)?	✓	✓	✓	✓	✓	✓	✓
Exposure measures and assessment	9. Were the exposure measures (independent variables) clearly defined, valid, reliable, and implemented consistently across all study participants?	✓	✓	✓	✓	✓	✓	✓
Repeated exposure assessment	10. Was the exposure(s) assessed more than once over time?	✓	✓	✓	✓	✓	✓	✓
Outcome measures	11. Were the outcome measures (dependent variables) clearly defined, valid, reliable, and implemented consistently across all study participants?	✓	✓	✓	✓	✓	✓	✓
Blinding of outcome assessors	12. Were the outcome assessors blinded to the exposure status of participants?	⦸	⦸	⦸	⦸	⦸	⦸	⦸
Follow up rates	13. Was loss to follow-up after baseline 20% or less?	✓	✓	✓	✓	✓	✓	✓
Statistical analysis	14. Were key potential confounding variables measured and adjusted statistically for their impact on the relationship between exposure(s) and outcome(s)?	✓	✓	✓	✓	✓	✓	✓
	*Total Points*	12	12	12	13	13	12	12
	Quality Rating	⬤	⬤	⬤	⬤	⬤	⬤	⬤

Depending on the information available in the publication, an ✓, ✗ or ⦸ is given to represent “Yes”, “No” or “Not Applied”, respectively. All results were considered of a good quality with a total score of >10 (green circle).

**Table 2:** Study characteristics of studies included in the current review.

Study Characteristics						
Study	Publication Information		Study Information			
	Year	Country	Recruitment Criteria	Sample Size	Study Age	Gender Size (M/F)
[15]	2019	China	Patients with stage II and III rectal cancers treated with pre-operative nCRT. Cases without genetic testing data were excluded due to poor DNA quality.	74	>50 (n = 42), ≤50 (n = 32)	52/22
[16]	2016	China	Rectal cancer patients before nCRT therapy. Patients with distant metastasis, declining surgery after nCRT and insufficient tumor tissue samples were excluded.	148	<65 (n = 67), ≥65 (n = 81)	89/59
[17]	2016	Italy	LARC patients undergoing homogeneous nCRT. Patients without sufficient quality of pre-therapy DNA were excluded.	108	Median = 67 (range: 31–80)	75/33
[18]	2015	Italy	LARC patients eligible for nCRT. Patients with incomplete CRT were excluded.	81	Mean = 63 (range: 44–91)	52/29
[19]	2014	Republic of Korea	First step: LARC patients who received preoperative CRT and curative resection were recruited for genome-wide methylation analysis by microarray (Cohort 1). Second step: consisted of a continuous cohort of LARC patients, including 27 patients from first step, with the same therapy regimen were added for validation by pyrosequencing (Cohort 2).	Cohort 1: 45 Cohort 2: 43	Mean = 59 Cohort 1 SD: ±12 Cohort 2 SD: ±11	Cohort 1: 34/11 Cohort 2: 29/14
[20]	2014	Spain	Patients treated with preoperative CRT for LARC (stages cT3–4 and/or N1–N2). Patients with metastases were excluded from the study	159	Mean = 65 (SD: 10.7) Median = 64 (range: 23–84)	104/55
[21]	2013	USA	Middle- or lower-third rectal cancer patients (stage II or III) who met the clinical criteria for nCRT and eventual hepatic mastectomy candidates with ultra-low stage I and stage IV progression.	33	Mean = 55.9 (SD: 10.51)	24/9

Data extracted and summarized from publication and study information.

**Table 3:** Clinical characteristics extracted from relevant studies.

Clinical Characteristics							
Study	Pathological Information			Treatment Protocol			Remark
	Pre-Therapeutic Clinical Staging (n)	Tumor Histological Grade (n)	Other Information	CT	RT	Surgical	
[15]	cTNM (AJCC 8th ed): cT2 (2), cT3 (31), cT4a (8), cT4b (33) cAJCC stage: II (25), III (49)	G1 (15), G2 (37), G3 (8)	Carcinoma Type: Adenocarcinoma (60), Mucinous carcinoma (12), Signet ring cell carcinoma (2)	XELOX and mFOLFOX-6 for 1 to 4 cycles before RT. Xeloda or 5-Fu concurrent with RT.	45 to 50.4 Gy over 28 fractions by IMRT or VMAT.	Curative resection, including TME at 4 to 8 weeks after completion of preoperative CRT.	Staging was evaluated by radiography and endoscopic ultrasonography, followed by surgical resection and IHC.
[16]	cTNM (AJCC 7th ed): cT3 (80), cT4 (68) cN0 (54), cN+ (94) M0 (122), M1 (26)	Differentiated (82), Undifferentiated (66)	CEA (ng/mL) <3.4 (70), ≥3.4 (78) Recurrence: Neg (105), Pos (43) Distance from anal verge (cm) <6 (57), ≥6 (91)	5-Fu or XELOX concurrent with RT.	50.4 Gy over 28 fractions with 4 fields of irradiation or 3D conformal RT	TME at 4 to 6 weeks after nCRT.	Biopsies were obtained by proctoscopy from patients before therapy to confirm the pathological diagnosis of adenocarcinoma within 15 cm from the anal verge. Physical examination, CEA routine blood test and chest enhancement CT, as well as abdomen and pelvic cavity enhancement CTs were performed before therapy.
[17]	uTNM: uT2 (8), uT3 (95), uT4 (3), uT N/A (2) uN0 (53), uN1 (31), uN2 (2), uNx (19), uN N/A (3)	N/A	N/A	Daily dose of 5-Fu during RT or Xeloda twice daily.	50.4 Gy over 5 weeks with conventional fractionation.	Surgery at 6 to 8 weeks after therapy completion.	Pre-therapeutic TNM staging was performed by ultrasound (uTNM).
[18]	N/A	G1 (4), G2 (66), G3 (11)	N/A	51 patients received standard Xeloda concurrent with RT, whilst 30 patients underwent XELOX	50.4 Gy in 28 fractions.	Surgery was performed 6 to 8 weeks after end of CRT.	The authors did not provide pre-therapeutic TNM staging.
[19]	AJCC 7th ed. stage II/III/IV: Cohort 1 (3/40/2), Cohort 2 (2/40/1)	4 patients in each cohort have poorly differentiated or mucinous tumor characteristics	CEA (ng/mL) Cohort 1: 4.57 ±6.25, Cohort 2: 13.91 ±52.29	Xeloda or 5-Fu delivered concurrently with RT	46 Gy delivered in 23 fractions followed by a 4 Gy boost to the primary tumor.	Radical surgery performed 6 to 8 weeks after CRT.	N/A
[20]	cTNM (AJCC 7th ed): cT0–2 (9), cT3–4 (150) cN+ (116)	G1 (16), G2 (134), G3 (9)	Tumor Location: Upper (43), Middle (61), Lower (54), Other (1)	Xeloda concomitant with RT For patients with adverse risk factors, 6 cycles of Xeloda or 6 cycles of XELOX or FOX was given after surgery	50.4 Gy delivered in 1.8 Gy fractions at 5 times per week by 3D conformal RT with a three-field technique	Radical surgery consisting of APR or LAR was performed 4 to 6 weeks after CRT.	N/A
[21]	NCCN stage: I (2), II (11), III (14), IV (4), N/A (2)	Differentiation Well (2), Moderate (29), Poor (1)	Invasion: Vascular (1), Lymphatic (5)	5-Fu delivered as radiosensitizer	5040 [c]Gy delivered in 30 fractions	Proctectomy performed approximately 8 to 10 weeks after CRT	Initially, the author reported a 5040 Gy radiation dose, but after confirmation with experts, the dose unit should be cGy (100 cGy = 1 Gy). This could be a minor typo by the literature.

The pre-therapeutic staging, tumor histological type and other pathological information was extracted along with the treatment protocol and other clinical remarks. IMRT—intensity-modulated radiation therapy, VMAT—volumetric-modulated arc radiotherapy, APR—abdominoperineal resection, and LAR—lower anterior resection.

**Table 4:** Outcome measures detailing tumor responses and mutation information were extracted from eligible studies.

Outcome Characteristics						
Study	Tumor Response			Mutation Information		Remark
	TRG Criteria	Grade (n)	Post-Therapeutic Pathological Staging (n)	*Gene* (Mutation Type) [Frequency (n)]	Detection Method	
[15]	NCCN	0 (13), 1 (19), 2 (30), 3 (12)	ypTNM: ypT0 (13), ypT1 (1), ypT2 (14), ypT3 (38), pT4a (2), ypT4b (6) Downstaging observed (50) ypAJCC_stage: 0 (12), I (11), II (24), III (25), Uncertain (2)	*BRAF* [TRG2 (1), TRG3 (3)]; *RAF1* [TRG2 (1)]; *KRAS* [TRG0 (4), TRG1 (6), TRG2 (12), TRG3 (4)]; *NRAS* [TRG2 (1)]; *TP53* [TRG0 (8), TRG1 (11), TRG2 (15), TRG3 (6)]; *APC* [TRG0 (3), TRG1 (4), TRG2 (4), TRG3 (3)]; *PIK3CA* [TRG2 (1)]; *PTEN* [TRG3 (1)]; *FBXW7* [TRG0 (2), TRG1 (2), TRG2 (3)]; *SMAD4* [TRG0 (1), TRG1 (1), TRG2 (2), TRG3 (4)]; *ERBB2* [TRG1 (1)]; *ERBB3* [TRG0 (1), TRG2 (2)]; *ATM* [TRG0 (1), TRG1 (2)]; *AKT1* [TRG0 (1), TRG3 (1)]; *RET* [TRG2 (1)]; *PDGFRA* [TRG0 (1)]; *SMARCB1* [TRG2 (2)]; *EZH2* [TRG0 (1)]; *CDKN2A* [TRG2 (1)]; *CTNNB1* [TRG2 (1)]; *EGFR* [TRG2 (1)]; *FLT3* [TRG2 (1)]; *JAK1* [TRG2 (1)]; *MPL* [TRG2 (1)]; *PTCH1*[TRG2 (1)]	Targeted NGS, Sanger sequencing, IHC	Mutation type was not studied in-depth. Downstaging is defined as tumor which has a lower ypT than cT. TRG grading represents the following post therapy tumor response: TRG0—complete response, TRG1—moderate response, TRG2—minimal response, TRG3—poor response.
[16]	Dworak	0–2 (79), 3–4 (69)	ypTNM: ypT0–2 (62), ypT3–4 (86) ypN0 (88), ypN+ (60) Downstaging observed (77)	*GOLPH3* (High) [TRG0–2 (49), TRG3–4 (28)], (Low) [TRG0–2 (30), TRG3–4 (41)]; *mTOR* (High) [in Highly expressed *GOLPH3* (53/77)], (Low) [in Low expressed *GOLPH3* (43/71)]	IHC	TRG0–2 was defined as poor response, whilst TRG3–4 indicates good response. TRG4 also represents pCR Studied protein expression levels rather than direct gene expression levels.
[17]	Dworak	0 (3), 1 (23), 2 (38), 3 (25), 4 (19)	ypTNM: ypT0 (19), ypT1 (11), ypT2 (31), ypT3 (39), ypT4 (4), ypT information not available (4) ypN0 (77), ypN1 (15), ypN2 (7), ypNx (3), ypN infomation not available (6)	*MIR17HG* cluster members (High levels of *miR-19a*, *miR-19b-1* and *miR-92a-1*) [TRG0–1 vs. TRG4 (100%)], (Locus amplified) [Non-responders (41%)], (Deletion) [Responders (41%)]; *CMYC; ABCC4*	RT-qPCR	Despite discovering upregulation of a miR levels, no statistical significance with any clinicopathological characteristics was found. No significances in *CMYC* and *ABCC4* expression with nCRT response were found. TRG0–1 and TRG4 denotes absence of response and complete response respectively.
[18]	Mandard	1 (20), 2 (16), 3 (22), 4 (21), 5 (2)	ypTNM: ypT0 (20), ypT1 (4), ypT2 (20), ypT3 (35), ypT4 (2) ypN0 (59), ypN1 (21) Downstaging observed (48)	*YKL-40*, (Positive) [TRG2–5 (87%)]; *c-Met*, (Positive) [TRG2–5 (86%)], (co-mutation with *YKL-40*) [TRG2–5 (94%)]	IHC, FISH	TRG1 describes complete response, whilst TRG2–5 indicates partial or absent response. Complete vs. partial responder distribution was similar for both the RT + capecitabine and XELOXART protocols. Disease status after therapy and surgery: No evidence of disease (n = 59), Alive with disease (n = 18), Died of disease (n = 3). Studied proteomic expression data.
[19]	Mandard	For each Cohort 1 against Cohort 2: 1 (7/12), 2 (12/11), 3 (18/12), 4 (8/8), 5 (0/0)	N/A	*KLHL34* CpG site: *cg14232291* (Hypermethylation) [levels in TRG1–3 = 42.45 ±3.21 vs. non-responders = 27.31 ±4.99]	Pyrosequencing, RT-qPCR, Western Blotting, Microarray	Staging of disease after therapy and radical surgery as well as frequency of mutation in patients was not reported. Positive response is assessed with TRG1–3.
[20]	N/A	N/A	ypTNM: ypT0N0 (29), ypT1–2N0 (99), ypT3–4 and/or pN+ (31)	Patients with ypT0N0 or ypT1–2N0 vs. ypT3–4 and/or pN+ *NFKB1* polymorphism: *rs28362491* (DEL/DEL) [(19) vs. (1)], (INS/INS) [(53) vs. (16)], (INS/DEL) [(56) vs. (14)]; *IL1B* polymorphism: *rs1143627* (A/A) [(61) vs. (9)], (G/A) [(47) vs. (17)], (G/G) [(20) vs. (5)]; *IL1B* polymorphism: *rs16944* (A/A) [(17) vs. (3)], (G/A) [(50) vs. (18)], (G/G) [(61) vs. (10)]; *PTGS1* polymorphism: *rs1213266*, (A/A) [(2) vs. (0)], (G/A) [(26) vs. (4)], (G/G) [(100) vs. (27)]; *PTGS1* polymorphism: *rs5789* (C/C) [(116) vs. (27)], (C/A) [(11) vs. (3)], (A/A) [(1) vs. (1)]; *PTGS2* polymorphism: *rs5275* (A/A) [(56) vs. (16)], (G/A) [(58) vs. (13)], (G/G) [(14) vs. (2)]	RT-qPCR	Directly reported mean differences in tumor size (cm) before and after treatment. Pre-treatment (by pelvic MRI): 6.26 (range: 1–12), Post-treatment (by pathological report): 2.26 (range: 0–1.85). Post-therapeutic response assessment was determined as follows: Complete response (ypT0N0), Intermediate or partial response (ypT1–2N0), Poor response (ypT3–4 and/or pN+) Vital status of patients: Deceased with tumor (n = 23), Deceased without tumor (n = 0), Alive with tumor (n = 7), Alive without tumor (n = 129).
[21]	AJCC	0 (6), 1 (7), 2 (13), 3 (7)	N/A	Top 10 (up-regulated) genes: *APOA2*, *AHSG*, *DBH*, *APOA1*, *APOB*, *APOC3*, *LMX1A*, *SOAT2*, *SLC7A9*, *TF* Top 10 (down-regulated) genes: *LOC729399*, *SERINC5*, *SCNN1B*, *ZC3H6*, *SLC4A4*, *DTWD2*, *MS4A12*, *BEX5*, *MMRN1*, *CLCA4*	Microarray	AJCC0–2 were considered responders AJCC3 were considered non-responders. Staging of disease after therapy and radical surgery as well as mutation frequency was not reported. Only reported top 10 up- and down-regulated genes out of 19, 228 target genes for non-responders.

For easy illustration, the gene of interest are shown in italic font, whilst the type of mutation and frequency of mutation were denoted in round and square brackets, respectively.

**Table 5:** List of identified genes which were involved in relevant pathways associated to cancer radioresponse.

Gene Set Enrichment Analysis Summary				
	Apoptosis	DNA Damage Response and Repair	Inflammation	Cancer Metabolism
*AKT1*	✓	✓	✓	✓
*APC*		✓		
*ATM*	✓			
*BRAF*			✓	
*CDKN2A*	✓	✓		
*CTNNB1*		✓		
*EGFR*			✓	✓
*ERBB2*		✓		✓
*FLT3*				✓
*KRAS*	✓	✓	✓	✓
*MET*				✓
*mTOR*				✓
*MYC*		✓		✓
*NFKB1*	✓	✓		
*NRAS*	✓	✓	✓	✓
*PDGFRA*				✓
*PIK3CA*	✓	✓	✓	✓
*PTEN*		✓	✓	✓
*PTGS1*			✓	
*PTGS2*			✓	
*RAF1*	✓		✓	✓
*RET*				✓
*SMAD4*		✓		
*TP53*	✓			✓

✓ represent “Yes”, this gene participates in the pathway

## Data Availability

The original contributions presented in the study are included in the article/supplementary material, further inquiries can be directed to the corresponding author/s.

## Supplementary Materials

The following supporting information can be downloaded at: https://www.mdpi.com/article/10.3390/genes15101257/s1, Figure S1: PRISMA Flowchart; Table S1: Gene molecular mechanism and CRC radioresponse; Table S2: NCBI gene search results.
