# Supplementary material for: Molecular Mechanism of Radioresponsiveness in Colorectal Cancer: A Systematic Review
Source: Genes (Basel). 2024 Sep 26;15(10):1257. doi: 10.3390/genes15101257 (PMC11508137; doi:10.3390/genes15101257)
Supplement: Supplementary file 1 [file genes-15-01257-s001.zip › Table S1 - Gene molecular mechanism and CRC radioresponse.pdf]

Table S1

Gene molecular mechanism and CRC radioresponse

Below table details the influence of the molecular mechanism of each identified gene to CRC radioresponse according to TRG grading.

Study	Gene	Molecular Mechanism	CRC radioresponse by TRG
[15]	*BRAF*	Point mutation (c.1799T>A; p.Val600Glu)	Variable response
		Point mutation (c.1781A>G; p.Asp594Gly)	Poor response
	RAF1	Point mutation (c.770C>T; p.Ser257Leu)	Minimal response
	KRAS	Point mutation (c.183A>T; p.Gln61His)	Variable response
		Point mutation (c.34G>A; p.Gly12Ser)	Minimal response
		Point mutation (c.34G>T; p.Gly12Cys)	Variable response
		Point mutation (c.35G>A; p.Gly12Asp)	Variable response
		Point mutation (c.35G>C; p.Gly12Ala)	Moderate response
		Point mutation (c.35G>T; p.Gly12Val)	Variable response
		Point mutation (c.38G>A; p.Gly13Glu)	Variable response
		Point mutation (c.436G>A; p.Ala146Thr)	Minimal response
		Point mutation (c.64C>A; p.Gln22Lys)	Poor response
	NRAS	Point mutation (c.183A>C; p.Gln61His)	Minimal response
	TP53	Point mutation (c.1024C>T; p.Arg342Ter)	Variable response
		Point mutation (c.144C>T; p.Asp48=)	Minimal response
		Intronic point mutation (c.375+1G>A; p.?)	Minimal response
		Intronic point mutation (c.375+5G>T; p.?)	Minimal response
		Point mutation (c.375G>A; p.Thr125=)	Moderate response
		Point mutation (c.467G>A; p.Arg156His)	Good response
		Point mutation (c.499C>T; p.Gln167Ter)	Minimal response
		Point mutation (c.524G>A; p.Arg175His)	Variable response
		Point mutation (c.526T>G; p.Cys176Gly)	Minimal response
		Point mutation (c.536A>G; p.His179Arg)	Poor response
		Point mutation (c.584T>C; p.Ile195Thr)	Moderate response

		Point mutation (c.586C>T; p.Arg196Ter)	Good response
		Deletion (c.626_627delGA; p.Arg209fsTer6)	Good response
		Point mutation (c.637C>T; p.Arg213Ter)	Poor response
		Point mutation (c.638G>T; p.Arg213Leu)	Moderate response
		Deletion (c.686_687delGT; p.Cys229fsTer10)	Moderate response
		Point mutation (c.701A>G; p.Tyr234Cys)	Moderate response
		Point mutation (c.733G>A; p.Gly245Ser)	Moderate response
		Point mutation (c.742C>T; p.Arg248Trp)	Poor response
		Point mutation (c.743G>A; p.Arg248Gln)	Variable response
		Point mutation (c.746G>T; p.Arg249Met)	Minimal response
		Intronic point mutation (c.783-1G>A; p.?)	Poor response
		Point mutation (c.809T>G; p.Phe270Cys)	Minimal response
		Point mutation (c.817C>T; p.Arg273Cys)	Variable response
		Point mutation (c.832C>T; p.Pro278Ser)	Moderate response
		Point mutation (c.833C>G; p.Pro278Arg)	Moderate response
		Point mutation (c.844C>T; p.Arg282Trp)	Minimal response
		Point mutation (c.853G>A; p.Glu285Lys)	Minimal response
		Point mutation (c.938G>A; p.Ser313Asn)	Minimal response
	APC	Point mutation (c.1983T>C; p.Cys661=)	Moderate response
		Point mutation (c.3916G>T; p.Glu1306Ter)	Minimal response
		Deletion (c.3921_3925delAAAAG; p.Glu1309fsTer4)	Variable response
		Point mutation (c.4128T>A; p.Tyr1376Ter)	Minimal response
		Point mutation (c.4132C>T; p.Gln1378Ter)	Good response
		Point mutation (c.4135G>T; p.Glu1379Ter)	Good response
		Point mutation (c.4216C>T; p.Gln1406Ter)	Minimal response
		Point mutation (c.4348C>T; p.Arg1450Ter)	Variable response
		Deletion (c.4385_4386delAG; p.Ser1465fsTer3)	Poor response
	PIK3CA	Point mutation (c.1258T>C; p.Cys420Arg)	Good response
		Point mutation (c.1035T>A; p.Asn345Lys)	Moderate response
		Point mutation (c.1624G>A; p.Glu542Lys)	Moderate response
		Point mutation (c.310C>A; p.Pro104Thr)	Minimal response
		Point mutation (c.1031T>C; p.Val344Ala)	Minimal response

		Point mutation (c.1633G>A; p.Glu545Lys)	Minimal response
		Point mutation (c.2177A>G; p.Glu726Gly)	Poor response
		Deletion (c.325_327delGAA; p.Glu109del)	Poor response
		Point mutation (c.3140A>G; p.His1047Arg)	Poor response
	PTEN	Point mutation (c.98T>G; p.Ile33Ser)	Poor response
	FBXW7	Point mutation (c.1268G>T; pGly423Val)	Minimal response
		Point mutation (c.1745C>T; p.Ser582Leu)	Minimal response
		Point mutation (c.2065C>T; p.Arg689Trp)	Good response
		Point mutation (c.796C>T; p.Arg266Cys)	Moderate response
		Point mutation (c.832C>T; p.Arg278Ter)	Variable response
	SMAD4	Point mutation (c.1051G>A; p.Asp351Asn)	Minimal response
		Point mutation (c.1052A>G; p.Asp351Gly)	Poor response
		Point mutation (c.1064A>G; p.Asp355Gly)	Moderate response
		Point mutation (c.1081C>T; p.Arg361Cys)	Poor response
		Point mutation (c.1082G>A; p.Arg361His)	Variable response
		Point mutation (c.1094G>A; p.Gly365Asp)	Minimal response
		Point mutation (c.1333C>T; p.Arg445Ter)	Minimal response
		Point mutation (c.1069G>T; p.Asp537Tyr)	Poor response
	ERBB2	Point mutation (c.2264T>C; p.Leu755Ser)	Moderate response
	ERBB3	Point mutation (c.785C>A; p.Pro262His)	Good response
		Point mutation (c.1331G>A; p.Arg444Gln)	Minimal response
		Point mutation (c.310G>A; p.Val104Met)	Minimal response
	ATM	Point mutation (c.8545C>T; p.Arg2849Ter)	Good response
		Point mutation (c.8774G>T; p.Gly2925Val)	Moderate response
		Point mutation (c.8122G>A; p.Asp2708Asn)	Moderate response
	AKT1	Point mutation (c.49G>A; p.Glu17Lys)	Variable response
	RET	Point mutation (c.1920C>T; p.Ala640=)	Minimal response
	PDGFRA	Point mutation (c.2228T>C; p.Val743Ala)	Good response
	SMARCB1	Point mutation (c.618G>A; p.Trp260Ter)	Minimal response
		Point mutation (c.601C>T; p.Arg201Ter)	Minimal response
	EZH2	Point mutation (c.507A>C; p.Glu169Asp)	Good response
	CDKN2A	Point mutation (c.134G>A; p.Gly45Asp)	Minimal response

	CTNNB1	Point mutation (c.93G>A; p.Leu31=)	Minimal response
	EGFR	Point mutation (c2618G>A; p.Gly873Glu)	Minimal response
	FLT3	Point mutation (c.1964G>A; p.Arg655Lys)	Minimal response
	JAK1	Point mutation (c.2170C>T; p.Arg724Cys)	Minimal response
		Point mutation (c.1930C>T; p.Gln644Ter)	Minimal response
	MPL	Point mutation (c.15229T>C; p.Leu510Pro)	Minimal response
	PTCH1	Point mutation (c.3241G>A; p.Val1081Met)	Minimal response
[16]	*GOLPH3*	High expression	Poor response
	mTOR	Correlate expression with *GOLPH3*	Poor response
[17]	*MIR17HG*	High levels of *miR-19a*, *miR-19b-1* and *miR-92a-1*	Poor response
		Copy number alterations	Variable response
	CMYC	Copy number alterations	No significant effect
	ABCC4	Copy number alterations	No significant effect
[18]	*YKL-40*	High protein expression	Poor response
	c-Met	High protein expression	Poor response
[19]	*KLHL34*	CpG site hypermethylation at *cg14232291*	Variable response
[20]	*NFKB1*	Homozygous deletion of polymorphism *rs288362491*	Variable response
		Hemizygous deletion of polymorphism *rs288362491*	Variable response
		Homozygous insertion of polymorphism *rs288362491*	Variable response
	IL1B	Homozygous A/A of polymorphism *rs1143627*	Variable response
		Hemizygous G/A of polymorphism *rs1143627*	Variable response
		Homozygous G/G of polymorphism *rs1143627*	Variable response
		Homozygous A/A of polymorphism *rs16944*	Variable response
		Hemizygous G/A of polymorphism *rs16944*	Variable response
		Homozygous G/G of polymorphism *rs16944*	Variable response
	PTGS1	Homozygous A/A of polymorphism *rs1213266*	Good response
		Hemizygous G/A of polymorphism *rs1213266*	Variable response
		Homozygous G/G of polymorphism *rs1213266*	Variable response
		Homozygous A/A of polymorphism *rs5789*	Variable response
		Hemizygous C/A of polymorphism *rs5789*	Variable response
		Homozygous C/C of polymorphism *rs5789*	Variable response
	PTGS2	Homozygous A/A of polymorphism *rs5725*	Variable response

		Hemizygous G/A of polymorphism *rs5725*	Variable response
		Homozygous G/G of polymorphism *rs5725*	Variable response
[21]	*APOA2*	Up-regulation	Poor response
	AHSG	Up-regulation	Poor response
	DBH	Up-regulation	Poor response
	APOA1	Up-regulation	Poor response
	APOB	Up-regulation	Poor response
	APOC3	Up-regulation	Poor response
	LMX1A	Up-regulation	Poor response
	SOAT2	Up-regulation	Poor response
	SLC7A9	Up-regulation	Poor response
	TF	Up-regulation	Poor response
	LOC729399	Down-regulation	Poor response
	SERINC5	Down-regulation	Poor response
	SCNN1B	Down-regulation	Poor response
	ZC3H6	Down-regulation	Poor response
	SLC4A4	Down-regulation	Poor response
	DTWD2	Down-regulation	Poor response
	MS4A12	Down-regulation	Poor response
	BEX5	Down-regulation	Poor response
	MMRN1	Down-regulation	Poor response
	CLCA4	Down-regulation	Poor response
