# Supplementary material for: Molecular Mechanism of Radioresponsiveness in Colorectal Cancer: A Systematic Review
Source: Genes (Basel). 2024 Sep 26;15(10):1257. doi: 10.3390/genes15101257 (PMC11508137; doi:10.3390/genes15101257)
Supplement: Supplementary file 1 [file genes-15-01257-s001.zip › Table S2 - NCBI gene results.pdf]

Table S2

NCBI gene search results

Below table outlined basic information from NCBI gene search of the 56 genes of interests extracted in the current study.

Study	Gene abbreviation	ENSG ID	Full gene nomenclature	Chromosome location	Function from NCBI gene search
[15]	*BRAF*	ENSG00000157764	B-Raf proto-oncogene, serine/threonine kinase	7q34	MAP kinase/ERK signaling pathway regulation.
	RAF1	ENSG00000132155	Raf-1 proto-oncogene, serine/threonine kinase	3p25.2	Encodes MAP3K.
	KRAS	ENSG00000133703	KRAS proto-oncogene, GTPase	12p12.1	Encodes a protein that is a GTPase superfamily member.
	NRAS	ENSG00000213281	NRAS proto-oncogene, GTPase	1p13.2	Encodes a membrane protein which shuttles between the Golgi apparatus and plasma membrane.
	TP53	ENSG00000141510	Tumor protein p53	17p13.1	Tumor suppressor protein which regulates gene expressions under cellular stresses.
	APC	ENSG00000134982	APC regulator of WNT signaling pathway	5q22.2	Encodes a tumor suppressor protein acting as an antagonist

					of the Wnt signaling pathway.
	PIK3CA	ENSG00000121879	Phosphatidylinositol-4,5-bisphosphate 3-kinase catalytic subunit alpha	3q26.32	Phosphorylation of PtdIns, PtdIns4P and PtdIns(4,5)P2.
	PTEN	ENSG00000171862	Phosphatase and tensin homolog	10q23.31	Tumor suppressor by negatively regulating AKT/PKB signaling pathway.
	FBXW7	ENSG00000109670	F-box and WD repeat domain containing 7	4q31.3	Encodes a F-box protein functioning in phosphorylation-dependent ubiquitination.
	SMAD4	ENSG00000141646	SMAD family member 4	18q21.2	A member of the Smad family of signal transduction protein acting as a tumor suppressor.
	ERBB2	ENSG00000141736	Erb-b2 receptor tyrosine kinase 2	17q12	Encodes a member of the EGF receptor family of receptor tyrosine kinase.
	ERBB3	ENSG00000065361	Erb-b2 receptor tyrosine kinase 3	12q13.2	Encodes a member of the EGF receptor family of receptor tyrosine kinase.
	ATM	ENSG00000149311	ATM serine/threonine kinase	11q22.3	Encodes a protein of the PI3/PI4-kinase family and is a important cell cycle checkpoint kinase

					which undergo phosphorylation.
	AKT1	ENSG00000142208	AKT serine/threonine kinase 1	14q32.33	Encodes one of the three members of the human AKT serine-threonine protein kinase family.
	RET	ENSG00000165731	Ret proto-oncogene	10q11.21	A transmembrane receptor and member of the tyrosine protein kinase protein family
	PDGFRA	ENSG00000134853	Platelet derived growth factor receptor alpha	4q12	A cell surface tyrosine kinase receptor for members of the platelet-derived growth factor family.
	SMARCB1	ENSG00000099956	SWI/SNF related, matrix associated, actin dependent regulator of chromatin, subfamily b, member 1	22q11.23	Relieves repressive chromatin structures, allowing transcription to occur.
	EZH2	ENSG00000106462	Enhancer of zeste 2 polycomb repressive complex 2 subunit	7q36.1	A member of the PcG gamily involved in maintaining transcriptional repressive states of genes over successive cell generations.
	CDKN2A	ENSG00000147889	Cyclin dependent kinase inhibitor 2A	9p21.3	Tumor suppressor functions.
	CTNNB1	ENSG00000168036	Catenin beta 1	3p22.1	Constitutes to adherens junctions

					necessary for the creation and maintenance of epithelial cell layers by cell growth regulation and cell adhesions.
	EGFR	ENSG00000146648	Epidermal growth factor receptor	7p11.2	A transmembrane glycoprotein for family members of the epidermal growth factor ultimately regulating cell proliferation.
	FLT3	ENSG00000122025	Fms related receptor tyrosine kinase 3	13q12.2	Regulates hematopoiesis.
	JAK1	ENSG00000162434	Janus kinase 1	1p31.3	Regulates genes that mediate inflammation, epithelial remodeling and metastatic cancer progression.
	MPL	ENSG00000117400	MPL proto-oncogene, thrombopoietin receptor	1p34.2	Major regulator of megakaryocytopoiesis and platelet formation.
	PTCH1	ENSG00000185920	Patched 1	9q22.32	A component of hedgehog signaling pathway.
[16]	*GOLPH3*	ENSG00000113384	Golgi phosphoprotein 3	5p13.3	A peripheral membrane protein of the Golgi apparatus and may contribute to Golgi trafficking.

	mTOR	ENSG00000198793	Mechanistic target of rapamycin kinase	1p36.22	Mediates cellular responses to stresses.
[17]	*MIR17HG*	ENSG00000215417	mIR-17-92a-1 cluster host gene	13q31.3	Host gene for the miR17-92 cluster consisting of atleast 6 miRNAs.
	CMYC	ENSG00000136997	MYC proto-oncogene, bHLH transcription factor	8q24.21	A proto-oncogene playing a role in cell cycle progression, apoptosis and cellular transformation.
	ABCC4	ENSG00000125257	ATP binding cassette subfamily C member 4	13q32.1	A member of the ATP binding cassette transporter superfamily.
[18]	*YKL-40*	ENSG00000133048	Chitinase 3 like 1	1q32.1	Role in inflammation and tissue remodeling process.
	c-Met	ENSG00000105976	MET proto-oncogene, receptor tyrosine kinase	7q31.2	Product of the proto-oncogene MET.
[19]	*KLHL34*	ENSG00000185915	Kelch like family member 34	Xp22.12	Encodes a protein located in extracellular space with unknown functions yet.
[20]	*NFKB1*	ENSG00000109320	Nuclear factor kappa B subunit 1	4q24	Role in inflammatory, cell growth and antiviral activity.
	IL1B	ENSG00000125538	Interleukin 1 Beta	2q14.1	Mediator of the inflammatory response and is involved in a number of cellular activities.

	PTGS1	ENSG00000095303	Prostaglandin-endoperoxide synthase 1	9q33.2	One of two genes encoding similar enzymes which catalyzes arachidonate conversion to prostaglandin, aswell as regulating angiogenesis in endothelial cells.
	PTGS2	ENSG00000073756	Prostaglandin-endoperoxide synthase 2	1q31.1	Encodes the inducible isozyme of PTGS and is key to prostaglandin biosynthesis and acts as dioxygenase and peroxidase.
[21]	*APOA2*	ENSG00000158874	Apolipoprotein A2	1q23.3	Second most abundant protein of the HDL particles.
	AHSG	ENSG00000145192	Alpha 2-HS glycoprotein	3q27.3	Suggestive roles in endocytosis, brain development and bone tissue formation.
	DBH	ENSG00000123454	Dopamine beta-hydroxylase	9q34.2	Catalyzes the conversion of dopamine to norepinephrine, expressed in the neurosecretory vesicles and chromaffin granules of the adrenal medulla.

	APOA1	ENSG00000118137	Apolipoprotein A1	11q23.3	Major protein component of HDL in plasma.
	APOB	ENSG00000084674	Apolipoprotein B	2q24.1	Main apolipoprotein of chylomicrons and LDL and is the ligand for the LDL receptor.
	APOC3	ENSG00000110245	Apolipoprotein C3	11q23.3	Encodes a protein component of TRLs including VLDL, HDL and chylomicrons.
	LMX1A	ENSG00000162761	LIM homeobox transcription factor 1 alpha	1q23.3	A transcription factor that as a positive regulator of insulin gene transcription.
	SOAT2	ENSG00000167780	Sterol O-acyltransferase 2	12q13.13	Encodes a membrane-bound enzyme responsible for intracellular cholesterol ester production from long-chain fatty acyl CoA and cholesterol.
	SLC7A9	ENSG00000021488	Solute carrier family 7 member 9	19q13.11	Encodes a protein playing a role in the transport of cystine and neutral and dibasic amino acids.
	TF	ENSG00000091513	Transferrin	3q22.1	Transport iron from the intestine, reticuloendothelial system and liver parenchymal cells to

					other proliferating cells in the body.
	LOC729399	None	Selenophosphate synthetase 1 pseudogene 6	2q33.1	N/A
	SERINC5	ENSG00000164300	Serine incorporator 5	5q14.1	Suggest to enable L-serine transmembrane transporter activities and viral defenses.
	SCNN1B	ENSG00000168447	Sodium channel epithelial 1 subunit beta	16p12.2	Encodes part of a sodium channel complex.
	ZC3H6	ENSG00000188177	Zinc finger CCCH-type containing 6	2q14.1	Suggest to enable RNA binding activity, metal ion binding activity and negative transcription regulation.
	SLC4A4	ENSG00000080493	Solute carrier family 4 member 4	4q13.3	Encodes an NBC involved in regulation of bicarbonate secretion, absorption and intracellular pH.
	DTWD2	ENSG00000169570	DTW domain containing 2	5q23.1	tRNA modification.
	MS4A12	ENSG00000071203	Membrane spanning 4-domains A12	11q12.2	Cell surface protein found primarily in the apical membrane of colonocytes with roles in proliferation, cell motility and chemotactic invasion

					of cancer cells in the colon.
	BEX5	ENSG00000184515	Brain expressed X-lined 5	Xq22.1	Predicted to be involved in signal transduction in the cytoplasm, enabling signaling receptor binding activities.
	MMRN1	ENSG00000138722	Multimerin 1	4q22.1	A large soluble protein found in platelets and in blood vessel endothelium.
	CLCA4	ENSG00000016602	Chloride channel accessory 4	1p22.3	Believed to encode a protein belonging to the calcium sensitive chloride conductance protein family.
