# Supplementary figures and images for: Molecular Mechanism of Radioresponsiveness in Colorectal Cancer: A Systematic Review
Source: Genes (Basel). 2024 Sep 26;15(10):1257. doi: 10.3390/genes15101257 (PMC11508137; doi:10.3390/genes15101257)

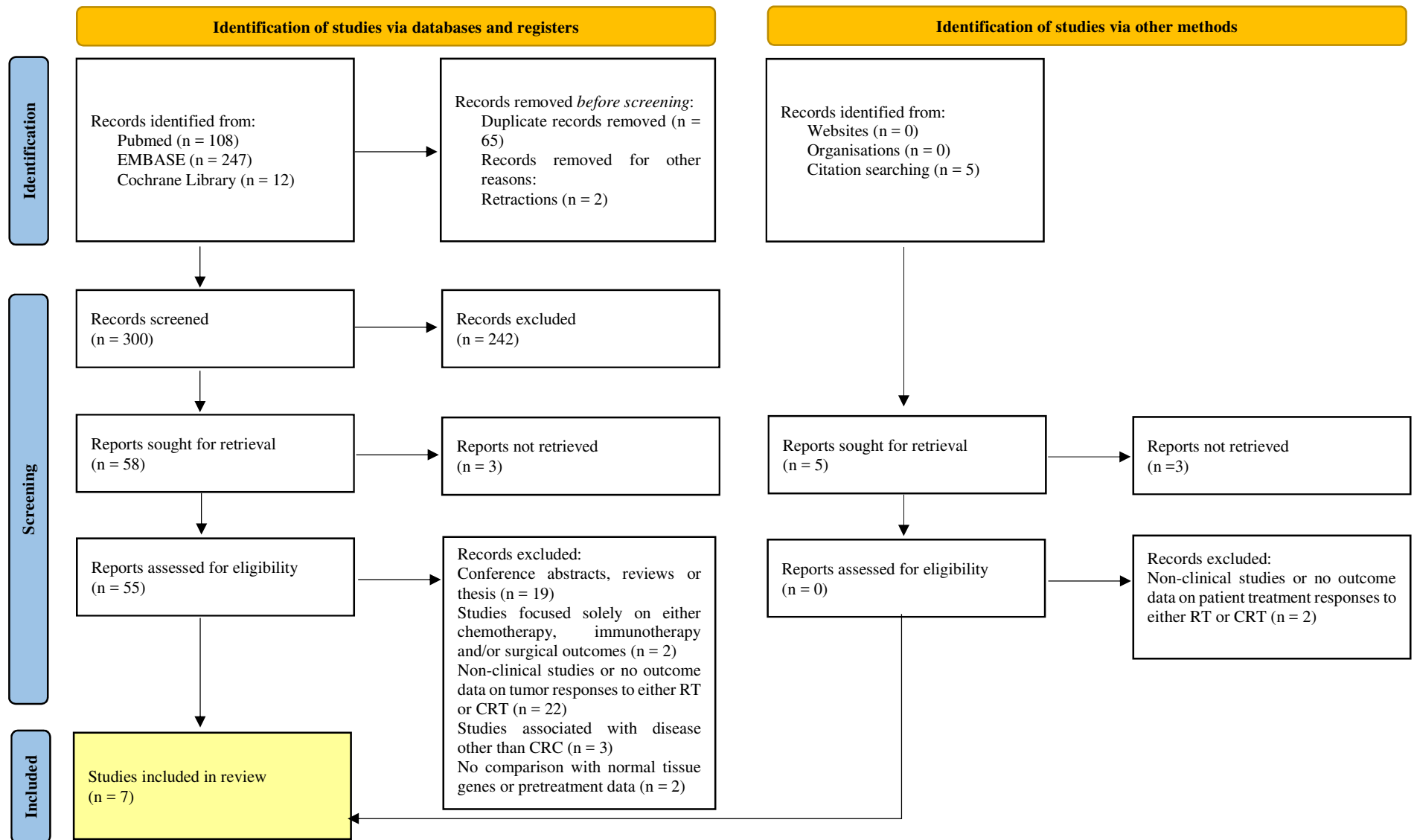

Figure S1. PRISMA Flowchart detailing search strategy and reason for study exclusions.

Supplement: Supplementary file 1 [file genes-15-01257-s001.zip › Figure S1 - Prisma Flow chart.pdf]
